# The effect of genistein on lipid levels and *LDLR, LXRα* and *ABCG1* expression in postmenopausal women with hyperlipidemia

**DOI:** 10.1186/s13098-019-0507-x

**Published:** 2019-12-19

**Authors:** Tao Zhang, Xiao-Xing Chi

**Affiliations:** 1 College of Medical Laboratory Science and Technology, Harbin Medical University-Daqing, Daqing, 163319 Heilongjiang Province China; 2 College of Food Science, Heilongjiang Bayi Agricultural University, Daqing, 163319 Heilongjiang Province China; 3 Agri-Food Processing and Engineering Technology Research Center of Heilongjiang Province, Daqing, 163319 Heilongjiang Province China

**Keywords:** Genistein, Postmenopausal women, Hyperlipidemia, Cholesterol related genes

## Abstract

**Background:**

This study investigates the effect of genistein (Gen) on the lipid profiles and expression of low-density lipoprotein receptor (LDLR), liver X receptor α (LXRα) and ATP-binding cassette transporter G1 (ABCG1) in the plasma macrophages of postmenopausal women with hyperlipidemia in China.

**Methods:**

This study considered 187 cases, where 160 postmenopausal women had hyperlipidemia. The subjects were divided into placebo group (PG) and experimental group (EG). EG received 60 mg/day of Gen, PG received placebo for 6 months. Body weight, height, waist circumference, body mass index and glucose levels were determined according to standard operating procedures. The triglyceride (TG), total cholesterol (TC), low density lipoprotein cholesterol (LDL-C), high density lipoprotein cholesterol (HDL-C), apolipoprotein-A1 (Apo-A1) and apolipoprotein-B (Apo-B) levels were detected in the plasma macrophages using ELISA. The protein and mRNA expression levels of LDLR, LXRα and ABCG1 were detected by western blot and real-time PCR techniques, respectively.

**Results:**

Compared to the baseline, Gen effectively lowered TG, TC and LDL-C levels, whereas HDL-C levels as well as the protein and mRNA expression levels of LDLR, LXRα and ABCG1 (*p* < 0.05) were increased. There was a significant difference in the expression of LDLR protein between the two groups (*p* < 0.05). The mRNA expression levels of LDLR, LXRα and ABCG1 were significantly increased in the EG compared to the PG.

**Conclusion:**

Gen effectively modulated the plasma lipid indices. The cholesterol-lowering effects of Gen may be attributed to its regulation on some of the key genes involved in cholesterol homeostasis.

## Background

High levels of cholesterol, atherosclerosis, coronary heart disease, and other cardiovascular diseases are increasingly prevalent and considered threats to human health [1, 2]. After menopause, total cholesterol (TC) and low-density lipoprotein cholesterol (LDL-C) usually increase, and these changes are frequently associated with changes in high-density lipoprotein cholesterol (HDL-C) and triglycerides (TG) [3]. In addition to the expected lipid abnormalities above, changes in the size and density of these lipoprotein particles are expected to occur after ovarian hormonal secretion is reduced [4].

Several proteins play a vital role in cholesterol metabolism. Low density lipoprotein receptor (LDLR) is a cell surface glycoprotein that plays an important role in the process of hepatic uptake and lipoprotein cholesterol cleaning. Increased LDLR expression or activity can reduce serum LDL cholesterol by enhancing the uptake and clearance [5]. ATP-binding cassette transporter G1 (ABCG1) is a key molecule for cholesterol efflux from macrophages and HDL biogenesis. ABCG1 facilitates cellular cholesterol efflux to lipidated particles such as mature HDL, but not to lipid-free apolipoproteins. The transcription of ABCG1 is under the control of liver X receptor (LXR), which is a major transcription factor important in cholesterol metabolism that is activated in response to cellular cholesterol levels 5 [6]. LXRα is belongs to the family of nuclear receptors and is one of the important factors that regulates many aspects of cholesterol homeostasis. These nuclear receptors are mainly distributed in the liver, adipose tissue, kidney, small intestine and macrophages. Their main physiological function is to control the cholesterol level, lipoprotein metabolism and fat synthesis [7]. The pathophysiological role of LXR α in humans is not fully understood.

Given the limitations of hormonal therapy [8, 9] the role of estrogen replacement has been limited to low-dose, short-term treatment. Based on the abovementioned studies, the search for alternative agents that may provide beneficial effects similar to estrogen while devoid of its adverse effects is warranted. After the consumption of isoflavones, complex enzymatic metabolic transformation occurs in the gastrointestinal tract, forming a heterocyclic phenol similar in structure to natural estrogen [10]. Synthetic estrogens and some anti-estrogens bind to estrogen receptors (ER) to initiate transcriptional activity [11]. Phytoestrogens are similar to estrogens and may exhibit antagonistic or antagonistic effects on ER based on the endogenous estrogenic environment.

Since several plants have been reported to have a cholesterol-lowering effect, novel plant preparations displaying lipid-lowering activity are being studied intensively. Genistein (Gen) is one of the most important active ingredients of isoflavone. The molecular structure of Gen is similar to estradiol. There are two phenolic hydroxyl residues in the bipolar relative polarity of estradiol, which can produce an estrogen-like effect when combined with estrogen receptors. A close relationship exists between the chemical structure and the biological activity of bioactive compounds. Therefore, the structural modification of Gen may alter its biological activity. A previous study showed that alkenylated Gen has antiestrogenic activity similar to 4-hydroxytamoxifen [12, 13]. In general, Gen is considered a phytoestrogen because it binds to trans-activated estrogen receptors and induces gene expression [14]. Gen efficacy was initially speculated to be based on its estrogen-like properties, and earlier research studies demonstrated that the chemically synthesized structural derivative of Gen, ipriflavone, exerted skeletal benefits [15]. In recent years, phytoestrogen supplements have been increasingly used as a safer alternative to estrogen, and their efficacy has been investigated in clinical trials [16, 17]. Previous clinical or human trials mainly studied the regulation effect of soybean isoflavone, soybean protein or soybean mixture on blood lipid levels [18–20]. Only animal trials have been performed on Gen. In this study, we investigated the effect of Gen, a monomer of soybean isoflavone, on lipid profiles. The precise mechanism by which Gen reduces serum and hepatic cholesterol has not been established. With regard to lipoproteins, a meta-analysis of 38 objects showed that soy supplementation results in an improvement in total and LDL-C [20], however, the benefit was mainly limited to subjects with very high pretreatment lipids. Furthermore, supplementation with 435 mg/day of isoflavone exerted a favorable effect on the total cholesterol, LDL-C levels and the ratio of apolipoprotein A1/apolipoprotein B in the blood of Chinese type 2 diabetic women [21]. On the basis of these studies, the regulatory effect and pathways of soybean bioactive substances on hyperlipidemic postmenopausal women were further investigated by measuring the protein and mRNA expression levels of LDLR, LXRα, and ABCG1 in hyperlipidemic postmenopausal women using western-blot and quantitative real-time polymerase chain reaction (qRT-PCR), respectively.

## Methods

### Study design and participants

One hundred and sixty participants including teachers from universities and colleges, hospital doctors, and thermal power plant workers who had a serum TC > 5.18 mmol/L or LDL-C > 3.37 mmol/L were included in the study. The study participant age ranged from 49 to 65 years, and the average age and body mass index were 56.9 ± 4.9 and 22.7 ± 0.8 kg/m^2^, respectively. The menopausal transition is characterized by variable cycle lengths and missed menses, whereas the postmenopausal period is marked by amenorrhea and thus these participants were selected. Women were excluded if they had any of the following: hepatic or renal disease, thyroid dysfunction, diabetes, neurological disease, active psychiatric disorder, history of breast cancer or abnormal mammograms, past or current history of chemotherapy and current use of glucocorticoids and serum triglycerides > 5.6 mmol/L. The participants considered for this study did not take lipid-lowering agents such as statins or ezetimibe for 6 months before the experiments. Before the trial, written informed consent was obtained from all the participants, and each participant volunteered to sign up to take genistein or placebo capsules in accordance with the strict schedule. The study was conducted at the CNC General Clinical Research Center where written informed consent was given prior to participation in this study. The study protocol was approved by the local and national ethics committees in accordance with the Good Clinical Practices Guidelines.

A list of randomized numbers was generated by a computer. One hundred and sixty female patients with hyperlipidemia were randomly assigned to the Gen intervention group or the placebo group. The participants and the personnel involved in the studies remained blinded to the group assignment throughout the study.

According to prior data, Gen given at 64 mg/day is associated with lowered HDL cholesterol levels compared to the placebo group1 [22]. Gen given at 54 mg/day for 12 months induced a 3% gain in BMD at the proximal femur and spine [23]. Based on these prior results, in this study a dose of 60 mg/day of Gen was used. The presence of approximately 60 mg of Gen in each capsule was confirmed by high-performance liquid chromatography (HPLC) analysis. The placebo powder contained the same nutrients as the intervention drug capsule (except for Gen). Both the experimental and placebo powders looked and tasted similar. The participants were asked to self-administer the capsules with food in the morning once a day for 6 months. Gen and placebo were provided by Heilongjiang Datong Company and supervised by Heilongjiang Society for Nutrition in China. The participants were instructed not to consume diets containing > 20 mg of soy isoflavone/day and instructed not to consume cholesterol-lowering agents. This study was conducted at the General Clinical Research Center of China National Centre.

The participant characteristics including body weight, height, waist circumference, and glucose levels were determined according to standard operating procedures. Two milliliters of 12 h-fasting venous blood was collected before and after the protocol. After the blood was centrifuged at 3000×*g* for 10 min at 4 °C, the supernatant was stored at − 80 °C until it was assayed. The blood glucose levels were analyzed using an enzymatic method (Bayer, Tokyo, Japan). Lipid parameters including triglycerides (Single reagent GPO-PAP), total cholesterol (Single reagent GPO-PAP), LDL-C (Bireagent direct method), HDL-C (Bireagent direct method), Apo-A1(ELISA) and Apo-B (ELISA) were measured using commercially available photometric test kits (Nanjing Jiancheng Bioengineering Institute, Nanjing, China). Human peripheral blood monocytes were isolated from the blood samples using Ficoll/Hypaque gradient centrifugation. The pooled monocytes were incubated in DMEM supplemented with 10% autologous serum for 10 days until they differentiated into macrophages [24].

### Quantitative real-time PCR for mRNA determination

Complementary DNA (cDNA) was synthesized with Reverse Transcriptase M-MLV (RNase H-) (TaKaRa, Dalian, China; Code No. D2639A) using the oligo dT. Real-time PCR technology was employed to determine the mRNA levels of *LDLR*, *LXRα* and *ABCG1* on the Light Cycler instrument (Roche Diagnostics, Germany) using the SYBR Green method. Each PCR mixture (final volume of 20 μL) was composed of 10 μL of SYBR qPCR Mix (TaKaRa, Dalian, China), 0.4 μL of each gene-specific primer, and 1 μL cDNA in each reaction. The primers used for real-time RT-PCR were as follows: LDLR: forward 5′-AGGAGTGCAAGACCAACGAG-3′—and reverse 5′-TACGTACCTCATGGCGGTTG-3′; ABCG1: forward 5′-CCTGTCTGATGGCCGCTTTC-3′ and reverse 5′-TCCCTCGGGTACGGAGTAAG-3′; LXRα: forward 5′-GAGTCATCCGAGCCTACAGC-3′ A and reverse 5′-AAGAATCCCTTGCAGCCCTC-3′ AGβ-actin: forward 5′-ACCCGCGAGTACAACCTTC-3′ and reverse 5′-ATGCCGTGTTCAATGGGGTA-3′. The thermal cycling parameters were as follows: 95 °C for 10 min, followed by 40 cycles of 95 °C for 15 s, 60 °C for 45 s, and 95 °C for 15 s, 60 °C for 1 min, 95 °C for 15 s, and 60 °C for 15 s. The relative expression of target genes was calculated using the 2^−ΔΔ*C*T^ method (the analysis was performed by the ABI Prism 7300 SDS Software).

### Western blot analysis for protein level determination

Cells were harvested and protein extracts prepared in accordance with the manufacturer’s instructions. The protein lysate (equal amount) from the cells was resolved by 12% sodium dodecyl sulfate polyacrylamide gel electrophoresis (SDS-PAGE) (Shanghai ShengGong Biological Technology Co. Ltd, Shanghai, China) and transferred to PVDF membranes (Millipore). The filters were blocked with TBST buffer containing 5% skim milk, and then incubated with primary antibodies LDLR (1:1000, rabbit), LXRα (1:1000, rabbit), and ABCG1 (1:1000, rabbit). Chemiluminescence (ECL) (Thermo Fisher) via the ImageQuant LAS 4000 mini (GE Healthcare Life Sciences, Shanghai, China) was used to detect the bands.

### Statististics

The data are shown as the mean ± SD. Differences in the mean percentage changes from baseline were evaluated by Student’s t test. The differences between groups were evaluated by Tukey test (*p* < 0.05) using SPSS 22.0. A p value of 0.05 or less was considered statistically significant.

## Results

Initially, 173 postmenopausal women were randomized, but the study was completed by 160 participants (EG, 77; PG, 83).

### Baseline characteristics of the study participants are shown in Tables 1 and 2

Both postmenopausal groups (PG and EG) were well matched the baseline without any significant differences in any of the characteristics (Table 1). The mean age, waist circumference, weight, height, BMI and glucose level were not significantly different between the two groups (*P *> 0.05). After treatment with Gen for 6 months, weight, body mass index and glucose levels decreased significantly (*P *< 0.05) in the EG (Table 2).

**Table 1:** Baseline general characteristics of women in different groups (mean ± SD)

General characteristics	PG	EG
Mean age (years old)	56.7 ± 4.9	57.2 ± 5.2
Waist circumference (cm)	81.1 ± 3.2	80.8 ± 3.1
Weight (kg)	60.2 ± 3.0	59.7 ± 2.4
Height (m)	162.2 ± 4.0	162.6 ± 3.0
BMI (kg/m^2^)	22.9 ± 0.9	22.6 ± 0.8
Glucose (mmol/L)	5.2 ± 0.7	5.4 ± 0.6

*PG* Placebo group, *EG* experiment group

Experimental conditions and treatment procedures are given in Materials and methods. Graph depicts (mean ± SD). Asterisk means significant against EG (*P < 0.05), t-test

**Table 2:** The change of general indicators of women 6 months later (mean ± SD)

		Baseline	6 months	^a^*p*	^b^*p*
Waist circumference (cm)	PG	81.1 ± 3.2	80.8 ± 2.8	0.147	0.118
	EG	80.8 ± 3.1	80.0 ± 3.5	0.109	
Weight (kg)	PG	60.2 ± 3	59 ± 1.4	0.182	0.061
	EG	59.7 ± 2.4	58.4 ± 2.3	0.002	
BMI	PG	22.9 ± 0.9	22.5 ± 1.2	0.186	0.067
	EG	22.6 ± 0.8	22.0 ± 0.8	0.002	
Glucose (mmol/L)	PG	5.2 ± 0.7	5.2 ± 0.7	0.165	0.038
	EG	5.4 ± 0.6	5.1 ± 0.6	0.004	

*PG* Placebo group, *EG* experiment group

Experimental conditions and treatment procedures are given in Materials and methods. Graph depicts (mean ± SD). ^a^*p* value denotes the comparison of mean changes from respective baseline between the experiment group and placebo group by two-sample t test. ^b^*p* value indicates the comparison of mean change from respective baseline between the experiment and placebo group were evaluated by Tukey test. *PG* Placebo group, *EG* experiment group

### The effect of genistein on lipid levels are shown in Table 3

As shown in Table 3, 6 months of Gen treatment significantly lowered TC (*P *= 0.0283), TG (*P *= 0.0006), LDL-C (*P *= 0.0015) and Apo-B (*P *= 0.0469) levels compared to baseline. Gen also raised the HDL-C levels (*P *= 0.0131) and the ratio of HDL/LDL (*P *= 0.0013). Furthermore, statistically significant differences for triglyceride, LDL-C, HDL-C and HDL/LDL levels were found between the EG and the PG (*p *< 0.05) at the end of the study. Apo-B levels decreased significantly in the EG compared to the PG (*p *< 0.05). No significant change was seen for any Apo-A1 in the two groups throughout the course of this study.

**Table 3:** The effect of genistein on lipid levels of women (mean ± SD)

Lipids		Baseline	6 months	^a^*p*	^b^*p*
Cholesterol (mmol/L)	PG	6.7 ± 1.1	6.6 ± 1.1	0.2108	0.199
	EG	6.7 ± 1.1	6.1 ± 1.1	0.0283	
Triglyceride (mmol/L)	PG	3.0 ± 1.1	3.0 ± 1.2	0.3382	0.001
	EG	3.0 ± 0.9	2.4 ± 1.1	0.0006	
HDL-C (mmol/L)	PG	1.1 ± 0.2	1.0 ± 0.3	0.2391	0.438
	EG	1.1 ± 0.1	1.2 ± 0.1	0.0131	
LDL-C (mmol/L)	PG	5.3 ± 1.1	5.3 ± 1.4	0.3912	0.000
	EG	5.2 ± 1.0	3.8 ± 0.6	0.0015	
HDL/LDL (mmol/L)	PG	0.2 ± 0.1	0.2 ± 0.1	0.3732	0.003
	EG	0.2 ± 0.1	0.3 ± 0.1	0.0013	
Apo-A1 (g/L)	PG	1.19 ± 0.04	1.18 ± 0.02	0.2117	0.438
	EG	1.19 ± 0.03	1.19 ± 0.02	0.4201	
Apo-B (g/L)	PG	1.01 ± 0.04	1.00 ± 0.03	0.2301	0.044
	EG	1.02 ± 0.04	0.68 ± 0.06	0.0469	

*PG* Placebo group, *EG* experiment group

Experimental conditions and treatment procedures are given in Materials and methods. Graph depicts (mean ± SD). ^a^*p* value denotes the comparison of mean changes from respective baseline between the experiment group and placebo group by two-sample t test. ^b^*p* value indicates the comparison of mean change from respective baseline between the experiment and placebo group were evaluated by Tukey test. *PG* Placebo group, *EG* experiment group

### The protein levels of LDLR, LXRα and ABCG1 in plasma macrophages are shown in Figs. 1 and 2

**Fig. 1:**
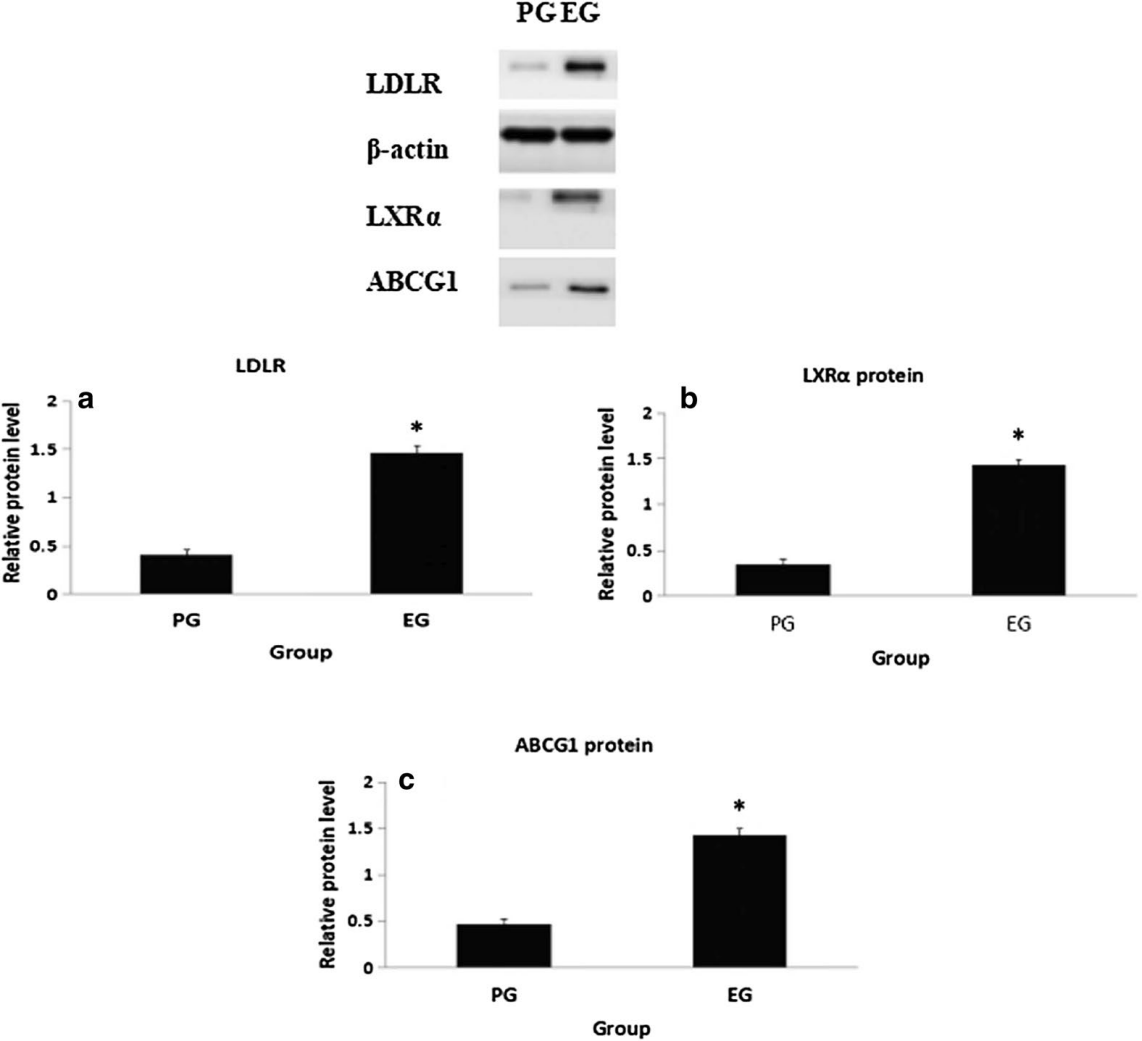
Protein expressions of LDLR (**a**), LXRα (**b**) and ABCG1 (**c**) in plasma macrophages after experiment measured by Western blot. Experimental conditions and treatment procedures are given in Materials and methods. Data are from post-experimental period measurements. Values are expressed as mean ± SD (n = 83.77). Values with different small letters differ significantly (*p *< 0.05). *Compared with placebo group PG

**Fig. 2:**
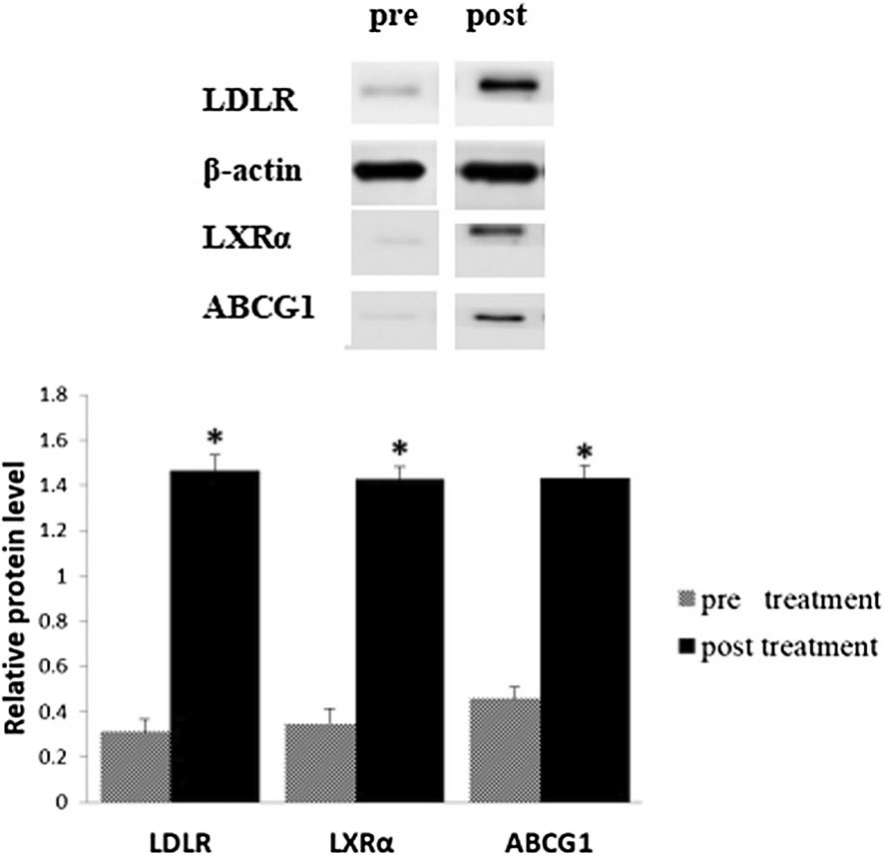
Protein expressions of LDLR, LXRα and ABCG1 in plasma macrophages in experimental group (EG) pre and post treatment with genistein measured by Western blot. Experimental conditions and treatment procedures are given in Materials and methods. Values are expressed as mean ± SD (n = 83,77). Values with different small letters differ significantly (*p *< 0.05). *Compared with the baseline

As shown in Fig. 1a, after 6 months of treatment, the protein level of LDLR in the EG was elevated compared with the PG (*p *< 0.05). The protein level of LDLR in the EG treated with Gen was greater than the PG (*p *< 0.01). Correspondingly, a change in the protein level of LXRα in both the groups was similar to LDLR (Fig. 1b). ABCG1 has been identified as a key player in regulating cellular cholesterol homeostasis. In this study, the influence of Gen on ABCG1 expression was observed. After treating with Gen (60 mg/day) for 6 months, an increase in the protein level of ABCG1 in the EG (Fig. 1c) was noted. Compared with baseline, the protein levels of LDLR, LXRα and ABCG1 were all increased in the EG (*p *< 0.05, Fig. 2).

### The mRNA expression levels of LDLR, LXRα and ABCG1 in plasma macrophages are shown in Figs. 3 and 4

**Fig. 3:**
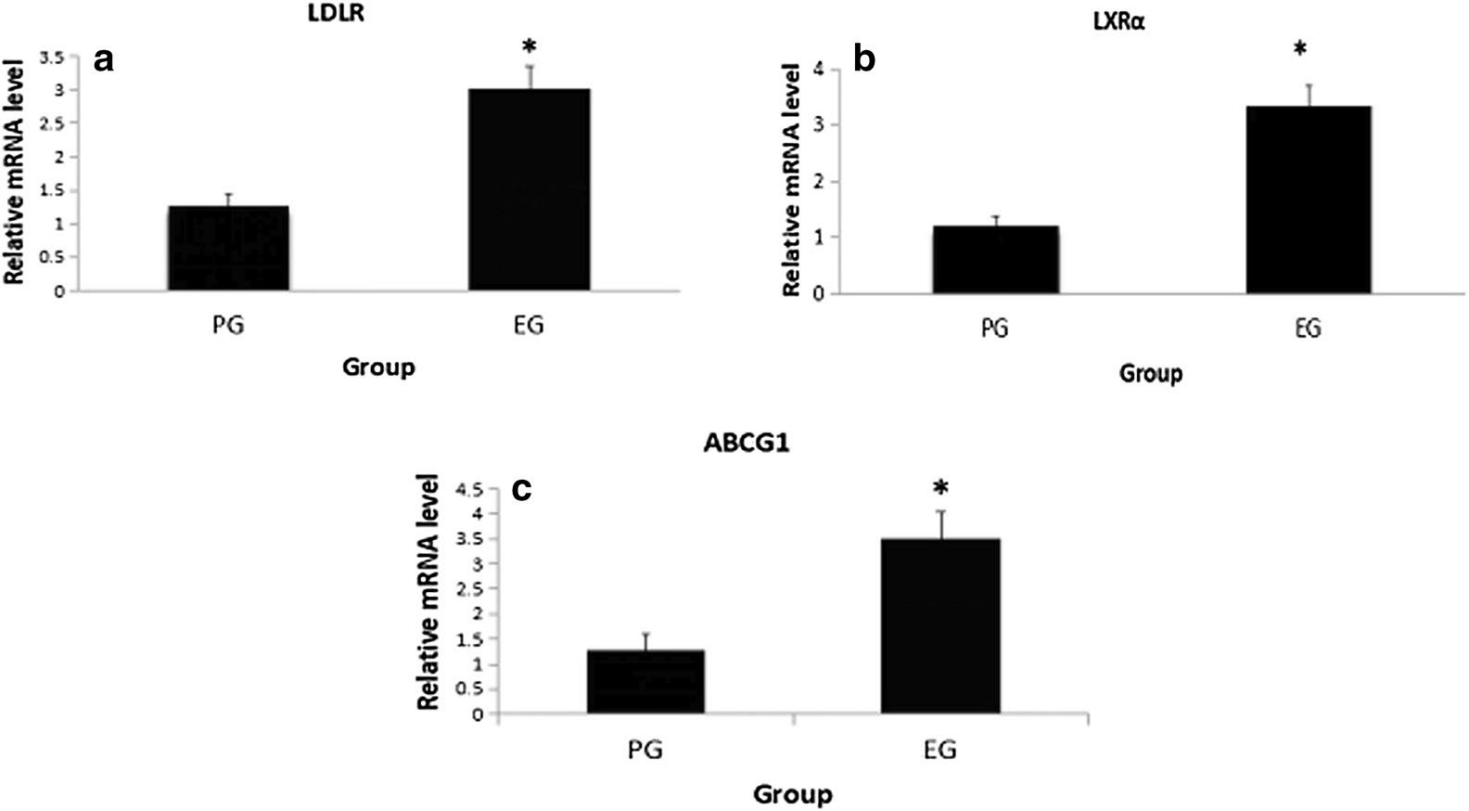
mRNA expressions of *LDLR* (**a**), *LXRα* (**b**) and *ABCG1* (**c**) in plasma macrophages measured by RT-PCR. Experimental conditions and treatment procedures are given in Materials and methods. Data are from post-experimental period measurements. Values are expressed as mean ± SD (n = 83, 77). Values with different small letters differ significantly (*p *< 0.05). *Compared with placebo group (PG)

**Fig. 4:**
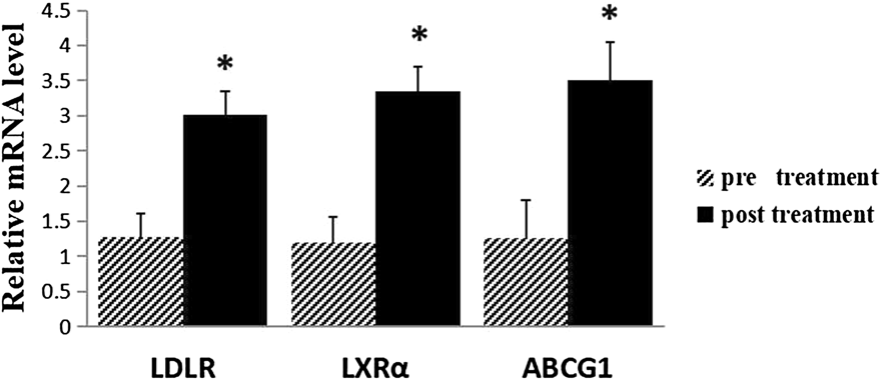
mRNA expressions of *LDLR*, *LXRα* and *ABCG1* in plasma macrophages in experimental group (EG) pre and post treatment with genistein measured by RT-PCR. Experimental conditions and treatment procedures are given in Materials and methods. Values are expressed as mean ± SD (n = 83, 77). Values with different small letters differ significantly (*p *< 0.05). *Compared with the baseline

As demonstrated in Fig. 3, the mRNA expression level of *LDLR* in the EG (relative mRNA level, 3.0) was increased markedly compared with the PG (relative mRNA level, 1.27) (*p *< 0.05). Compared with the PG (relative mRNA level, 1.19), the mRNA expression level of *LXRα* in the EG (relative mRNA level, 3.35) was significantly upregulated (*p *< 0.05, Fig. 3b). The effect of Gen on ABCG1 was similar to that observed with LXRα, which increases cholesterol efflux by inducing ABCG1. The mRNA expression level of *ABCG1* in the EG (relative mRNA level, 3.51) was significantly increased compared with the PG (relative mRNA level, 1.27) (*p *< 0.01, Fig. 3c). Additionally, the mRNA expression levels of LDLR, LXRα and ABCG1 in the plasma macrophages of the EG were significantly increased (*p *< 0.01, Fig. 4). The relative mRNA levels of LDLR, LXRα and ABCG1 increased from 1.27, 1.19 and 1.27 to 3.00, 3.35 and 3.51, respectively.

## Discussion

The present study clearly demonstrates that the consumption of Gen influences lipid levels and the expression of genes involved in the cholesterol reversal process of postmenopausal women with hyperlipidemia. The cholesterol lowering effect of soybean has been strongly associated with the estrogenic activity of isoflavones4 [25]. Moreover, the presence of isoflavones and Gen in soy protein were shown to be essential for reducing cholesterol levels and the development of atherosclerotic plaques in primates [26]. In this study, the role and mechanism underlying the effect of Gen on blood lipid levels were investigated.

Components associated with low-density lipoproteins, such as LDL-C and Apo-B, are independent risk factors for atherosclerosis. The clearance of LDLs in the plasma is regulated by a variety of cell surface receptors including LDLR. The plasma levels of LDL-c are largely regulated by LDLR, which mediates LDL clearance through a well-defined process involving endocytosis and degradation of whole LDL particles [27]. When the structure or function of LDLR is abnormal, plasma cholesterol levels are elevated. Plasma LDL-C may be regulated by upregulating the expression of LDLR by drugs or plant extracts, which may have therapeutic value [28]. In addition, soy protein has been shown to decrease the level of LDL-C by increasing LDLR [29]. In this study, compared with placebo, Gen significantly decreased the LDL-C level and upregulated the mRNA expression level of *LDLR*, indicating that it could reduce the LDL-C level by upregulating *LDLR*. These data agree well with the results of previous studies [30, 31]. An excessive elevation of Apo-B is closely related to the LDL-C level. In this study, the Apo-B level decreased after Gen treatment. In general, the concentration of Apo-A1 correlates with HDL-C; however, this study does not demonstrate a significant regulatory effect of Gen on Apo-A1.

LXRs are ligand-activated transcription factors that belong to the nuclear receptor superfamily. 40 Cholesterol flux between organs and cholesterol synthesis bile acids are ligands for certain nuclear receptors [32, 33]; however, whether the uptake of isoflavones or the monomeric Gen regulates the transcriptional control of LXRs remains unclear. LXRα can mediate the binding and transport factors of ABCG1 located in the small intestine of human macrophages to promote endogenous lipid membrane trafficking. ABCG1 is induced by LXR ligand, an important molecule that mediates phospholipid and cholesterol efflux, and plays an important role in the formation and metabolism of HDL-C [34]. After activation of LXR, the mRNA and protein expression levels of ABCG1 increased, and cholesterol outflow increased as well. LDLR, LXRα and ABCG1 are important indicators of the cholesterol reversal process. In this study, the effect of Gen on LDLR was explored in addition to changes in LXRα and ABCG1. Previous investigations indicated that in mammals, LDLR, LXRα and ABCG1 are abundantly expressed in macrophages [35]. Therefore, the levels of LDLR, LXRα and ABCG1 in the plasma macrophages were assessed. Compared with the PG, the protein and mRNA expression levels of *LXRα* in the EG were significantly higher. Gen significantly increased the expression of the LXR-responsive gene, ABCG1. Gen may first upregulate the expression of LXRα and then enhance the expression of ABCG1 resulting in decreased LDL-C and increased HDL-C in postmenopausal women. Furthermore, compared with baseline, the protein and mRNA expression levels of LDLR, LXRα and ABCG1 in the EG increased significantly after taking 6 months of Gen treatment. This result suggests that an improvement in the lipid levels following Gen treatment could be mediated by LDLR, LXRα and ABCG1. Thus, Gen could act as an LXR agonist to induce ABCG1 expression. Moreover, Gen enhanced the levels of LDLR, LXRα and ABCG1 proteins and genes in postmenopausal women with hyperlipidemia. Further studies are warranted to more fully understand the complete mechanism of action.

## Conclusion

The results from this investigation showed that the cholesterol-lowering effects of genistein may be attributed to its regulation of key genes involved in the metabolism of cholesterol, such as LDLR, LXR and ABCG1 in the plasma macrophages of hyperlipidemic postmenopausal women. This study also provides new evidence to further understand the mechanism of the development of hyperlipidemia and its prevention. Further studies are needed to understand the molecular mechanisms underlying the regulation of *LDLR, LXR* or other lipid-related gene isoforms by isoflavones or Gen.

## Data Availability

Data supporting the findings of this study are not publicly available, as the research is ongoing and further publications are being done.
